# Longitudinal trajectories of diet quality and subsequent mortality among Chinese adults: results from the China health and nutrition survey 1997–2015

**DOI:** 10.1186/s12966-021-01118-7

**Published:** 2021-04-07

**Authors:** Ming-wei Liu, Sarah A. McNaughton, Qi-qiang He, Rebecca Leech

**Affiliations:** 1 Julius Global Health. The Julius Center for Health Sciences and Primary Care, University Medical Center Utrecht, Utrecht, the Netherlands; 2 School of Health Sciences, Wuhan University, Wuhan, 430071 China; 3 Institute for Physical Activity and Nutrition (IPAN), School of Exercise and Nutrition Sciences, Deakin University, 221 Burwood Highway, Burwood, Victoria 3125 Australia; 4 Hubei Biomass-Resource Chemistry and Environmental Biotechnology Key Laboratory, Wuhan University, Wuhan, 430071 China

**Keywords:** Diet quality, Mortality, Cohort, Trajectory, China

## Abstract

**Background:**

China has witnessed a significant nutritional transition. However, there is a gap in the literature investigating the association between change of diet and mortality among Chinese. Thus, we aimed to explore the longitudinal trajectories of diet quality over 10 years (from 1997 to 2006) and the subsequent risk of death till 2015 among Chinese adults.

**Methods:**

Data from the China Health and Nutrition Survey were analyzed in 6398 adults. Dietary intake was assessed using three consecutive 24-h recalls. Diet quality was assessed by the Chinese Healthy Eating Index (CHEI), which includes 17 components and is based on the Dietary Guidelines for Chinese. Latent Class Growth Analysis was conducted to derive trajectories of diet quality over 10 years. Cox proportional hazard regression was used to calculate hazard ratios for total mortality.

**Results:**

Four distinct CHEI trajectories were identified: 1) worsening; 2) low-moderate-low; 3) improving; 4) high-moderate-high. Group 3 had the lowest mortality rate (5.6%) in the subsequent 9 years, while the groups with worsening or low diet quality had a higher mortality rate (Group 1: 7.5%; Group 2: 10.8%). In the fully adjusted model, compared to group 2, mortality rates were lower for group 3 (RR = 0.73; 95% CI: 0.55, 0.97) and group 4 (RR = 0.76; 95% CI: 0.59, 0.98). No associations with mortality were found for the group 1, when compared to group 2.

**Conclusions:**

Long-term improved diet quality and adherence to the Dietary Guidelines for Chinese may decrease the risk of death in Chinese adults.

**Supplementary Information:**

The online version contains supplementary material available at 10.1186/s12966-021-01118-7.

## Background

Fostering optimal diet to prevent chronic disease and mortality is a public health priority, globally and in China. In recent decades, a dietary pattern approach to understanding diet-disease relationships has been shown to better predict disease outcomes and mortality than approaches based on isolated nutrients or foods [1, 2]. This is because dietary patterns consider interrelationships and synergistic effects between multiple foods and nutrients [1]. Diet quality indices are well-established measures of dietary patterns. Compared to a data-driven or *posterior* approaches such as exploratory factor analysis, diet quality indices are theory-driven or a priori and assess the level of adherence to a healthy dietary pattern recommended by nutrition guidelines [1, 2]. Examination of dietary indices can help tailor guidelines to be more practical, because many combinations of foods can meet the recommendation, thus leaving people a flexible choice of foods [2].

Studies have shown that improved diet quality, as assessed by diet quality indices, is associated with a decreased risk of death in high-income countries or areas [3, 4]. A systematic review and meta-analysis combining results from 15 cohort studies showed that diets of the highest quality, as assessed by the Healthy Eating Index (HEI), the Alternative Healthy Eating Index (AHEI), and the Dietary Approaches to Stop Hypertension (DASH) score, was associated with a reduced overall mortality (risk reduction = 0.78; 95% CI = 0.76–0.80) [3]. Another meta-analysis of 18 cohort studies revealed that a 2-point increase in adherence score to the Mediterranean Diet was associated with a 9% reduction of overall mortality (95% CI = 7–11%) [4]. Given the strong evidence supporting their health benefits, these dietary patterns were recommended by the 2015 Dietary Guidelines for Americans [5].

However, evidence of associations between diet quality and mortality in low- and middle-income countries (LMIC), including China, is lacking. Wang et al. [6] assessed diet using AHEI among adults in 190 countries/territories with the Global Burden of Disease (GBD) Study, 2017, and found that improved diet quality predicted substantially lower global mortality. In the GBD Study, dietary data comprised few individual-level dietary surveys and the mortality burden was measured by the population attributable fraction (PAF) [6]. Further, the AHEI was based on the US dietary recommendations. More specific studies in LMICs based on their respective dietary guidelines are therefore needed.

In China, four dietary indices have been developed based on the Dietary Guidelines for Chinese (DGC) and the Chinese Food Pagoda (CFP) [7]. They are the Chinese Dietary Balance Index (DBI) [8], the Diet Quality Index (DQI) for China [9], the Chinese Food Pagoda Score (CFPS) [10], and the Chinese Healthy Eating Index (CHEI) [11, 12]. The DBI and DQI both used a bidirectional scoring system where components of overnutrition received positive scores and components of undernutrition received negative scores [8, 9]. The CFPS used a discrete scoring system ranging from 0 to 10 [10]. Based on the updated DGC-2016 [7], the newly developed CHEI used a continuous scoring system, which is easier to interpret and apply across diverse statistical analyses as compared to the bidirectional or discrete scoring systems [11]. However, these indices have rarely been applied to investigate their prospective associations with chronic disease or mortality in the Chinese population [11] and even less research has examined how health outcomes are affected by changes in long-term diet quality.

In the last two decades, China has witnessed a significant dietary transition, characterized by decreased fiber intake and increased animal meat intake [13, 14]. He et al. conducted a cross-sectional study to investigate the dietary transition of 11 individual food components and the diet-induced cardiometabolic mortality burden in 1982, 1992, 2002, and 2010–12, respectively [15]. However, the overall diet quality was not examined. Further longitudinal studies are needed to examine the association between change of diet quality and mortality. The sociodemographic disparity in the association between dietary transition and mortality is also of interest. To address these gaps, we utilized the CHEI to examine 10-year changes, or trajectories, in diet quality (from 1997 to 2006), and their associations with sociodemographic and subsequent mortality risk (from 2006 to 2015) among Chinese adults.

## Methods

### Study population

The China Health and Nutrition Survey (CHNS) is a large ongoing prospective study examining a wide range of information on economics, sociological issues, socio-demographics and health. To date, ten waves of surveys (1989 to 2015) have been conducted. A multistage, stratified sampling design was employed to select participants from nine provinces, accounting for about half of Chinese population. Detailed information about the cohort profile has been published previously [16]. The University of North Carolina at Chapel Hill, the Chinese Center for Disease Control and Prevention, the National Institute of Nutrition and Food Safety, and the China-Japan Friendship Hospital, Ministry of Health Institutional Review Committees approved the study protocol. All participants provided written informed consent.

The current study included seven waves of survey data from 1997 to 2015. Adults aged ≥20 years with complete dietary data, at least 3 waves of data between 1997 and 2006 and at least one wave of data after 2006 were eligible for inclusion (*n* = 6494). Participants who were ever pregnant during the survey (*n* = 65), and who were diagnosed with cardiovascular diseases or cancer (*n* = 31) were excluded. The final analytical sample included 6398 participants, the proportion of which in all adult participants of the CHNS 1997–2006 was 39.7%.

### Dietary assessment and the calculation of CHEI

The individual level of dietary intake was collected via 24-h recalls over three consecutive days (two weekdays and one weekend day) in each wave of the CHNS. Individually, each household member reported all foods consumed at home and away from home over the previous 24 h on each of the 3 days. Trained interviewers recorded types and amounts at each meal with the help of food models and pictures. Meanwhile, household food consumption was estimated by examining the daily changes of inventory using a weighing and measuring technique over the same three-day period. More detailed information is available elsewhere [17]. The Chinese Food Composition Tables [18–20] were used to categorize foods and to estimate nutrient intakes. The 1991 version [18] was used for dietary data 1997–2000 while the 2002 [19] and 2004 [20] versions were used for dietary data 2004–2011. The 2005 USDA database [21] was used to calculate added sugars due to the lack of available data in the Chinese Food Composition database. The individual’s intake of cooking oils, sodium, and added sugars were derived from objectively weighed household consumption data, and intake of other food components were derived from individual dietary data.

The CHEI was developed specifically for use in the Chinese population [11] and is based on the updated Dietary Guidelines for Chinese (DGC-2016) [7]. The CHEI has demonstrated good (content and construct) validity and internal consistency in the CHNS study population [12]. A detailed description of the methods used to calculate CHEI is provided elsewhere [11]. Briefly, standard portions (SP) for foods based on DGC-2016 were calculated [7, 11]. One SP of foods in a typical food group share consistent content of energy and similar macronutrient profiles [7]. Following this step, the previously developed CHEI scoring system for 17 components was applied [11]. As shown in Table 1, the CHEI comprises 12 adequacy components: total grains, whole grains and mixed beans, tubers, total vegetables, dark vegetables, fruits, dairy, soybeans, fish and seafood, poultry, eggs, and seeds and nuts, and 5 limitation/moderation components: red meat, cooking oil, sodium, added sugar, and alcohol. Components were scaled respectively from 0 to 5 (0 to 10 for fruits, cooking oils and sodium) with higher score indicating better diet quality [11]. Cutoffs for the minimum and maximum scores are converted based on DGC-2016 [7]. Scores for amounts between the cutoffs are prorated linearly. Total CHEI score is a sum of scores of all 17 components ranging from 0 to 100 (highest diet quality) [11].

**Table 1:** The scoring system of the Chinese Healthy Eating Index (CHEI)^1^

Component	Score^3^		
	0 (Min cutoff)	5 (Max cutoff)	10 (Max cutoff)^4^
Adequacy			
Total grains	0	≥2.5 SP/1000 kcal	/
Whole grains and mixed beans	0	≥0.6 SP/1000 kcal	/
Tubers	0	≥0.3 SP/1000 kcal	/
Total vegetables	0	≥1.9 SP/1000 kcal	/
Dark vegetables	0	≥0.9 SP/1000 kcal	/
Fruits	0		≥1.1 SP/1000 kcal
Dairy	0	≥0.5 SP/1000 kcal	/
Soybeans	0	≥0.4 SP/1000 kcal	/
Fish and seafood	0	≥0.6 SP/1000 kcal	/
Poultry	0	≥0.3 SP/1000 kcal	/
Eggs	0	≥0.5 SP/1000 kcal	/
Seeds and nuts	0	≥0.4 SP/1000 kcal	/
Limitation			
Red meat	≥3.5 SP/1000 kcal	≤0.4 SP/1000 kcal	/
Cooking oils	≥32.6 g/1000 kcal		≤15.6 g/1000 kcal
Sodium	≥3608 mg/1000 kcal		≤1000 mg/1000 kcal
Added sugars	≥20% of energy	≤10% of energy	/
Alcohol^2^	≥60 g (men) or ≥40 g (women)	≤25 g (men) or ≤15 g (women)	/

^1^Cite from: Yuan YQ, Li F, Dong RH, Chen JS, He GS, Li SG, et al. The development of a chinese healthy eating index and its application in the general population. Nutrients. 2017;9:1–18

^2^Children, adolescents, pregnant and lactating women who consumed alcohol get the score of zero for alcohol

^3^*SP* standardized portion. SP and the cutoffs were based on the Dietary Guidelines for Chinese (DGC-2016). Scores for amounts between the cutoffs were prorated linearly

^4^Ten is the maximum cutoff for fruits, cooking oils and sodium, while five is the maximum cutoff for other components

### Ascertainment of deaths

Person-years were calculated from the date of return of the 2006 questionnaire to the date of death or the end of follow-up (whichever came first). The mortality status along with exact date of death were ascertained by household information collected in each year. When a case of death was repeatedly reported, the initially reported date was used. Specific causes of death, however, were not reported in CHNS during 1997–2015.

### Assessment of covariates

All waves of surveys collected information about age, sex, residence, household income (inflated to 2015), marital status, physical activity (PA), smoke status, sleep duration (collected since 2004), height, weight, blood pressure (BP) measure, and diagnosis of hypertension and diabetes. Weight, height and BP were measured by trained technicians using standard method, and other covariates were self-reported. Weight was measured with participants in light clothing to the nearest 0.1 kg. Height was measured with participants being barefoot to the nearest 0.1 cm. The arterial BP was measured on the right arm using a standard mercury sphygmomanometer three times after sitting for 10 min. Residence was categorized as residing in urban or rural areas. Quantiles of change in household income were utilized. Change in marital status over time was categorized as: married to unmarried, unmarried (i.e. status unchanged), unmarried to married or married. Categories of change in meeting PA recommendations over time were: insufficiently active (no change), sufficiently to no longer sufficiently active, insufficiently to sufficiently active or sufficiently active. Sufficient activity was based on the World Health Organization’s (WHO) Global PA Recommendations [22]. Change in smoking status was categorized as: current smoker only, current smoker to former smoker, and not a current smoker. Sleep duration was categorized according to the US National Sleep Foundation’s recommendation as short, proper (7–9 h for adults and 7–8 h for old adults who aged ≥65 years) or long [23]. Body mass index (BMI) was computed as weight (in kg) divided by square of height (in m). History of hypertension was ascertained by meeting at least 1 of 4 criteria: (1) a new physician diagnosis of hypertension; (2) antihypertensive treatment; (3) systolic BP ≥ 140 mmHg; (4) diastolic BP ≥ 90 mmHg. History of diabetes was ascertained by self-reported diagnosis of diabetes.

### Statistical analysis

Latent Class Growth Analysis (LCGA) was used to classify groups of participants according to their diet quality trajectory over 10 years from 1997 to 2006. Quintiles of CHEI at years 1997, 2000, 2004 and 2006 were used as input variables. LCGA uses finite mixture modelling to estimate discrete groups of trajectory parameters estimated by maximum likelihood. The following steps were followed to determine the optimal growth model and number of latent longitudinal trajectories [24]. In a first step, a single latent growth model with linear and quadratic growth parameters were compared to see which approach best captured the trajectories’ growth. Following this, LCGA models comparing 1–6 classes were estimated. For each model convergence, a global maximum solution was found, as shown by replication of the largest log-likelihood value. The choice of the optimal model was based on a combination of statistical criteria, parsimony and interpretability [25]. Several model fit indices were used: (1) the minimum values of the goodness of fit measures Akaike’s information criteria (AIC), Bayes Information criteria (BIC); (2) the Vuong-Lo-Mendell-Rubin likelihood ratio test (VLMR LRT) for k versus k^− 1^ models; (3) the entropy value and the average posterior probability for each group [26, 27]. The meaningfulness of the trajectory shape and the number of participants in each group were also taken into consideration. Participants were assigned to the group for which they had the highest posterior probability of membership.

Comparisons of participants’ characteristics according to diet quality trajectories were tested using chi-square test for categorical variables and one-way ANOVA test for continuous variables. A comparison of the characteristics between included and excluded participants was also conducted (Supplemental Table 1 in Additional file 1). Proportions were used to describe changes in participants who meet the dietary recommendations in DGC-2016 for 17 food components of CHEI, respectively. Cox proportional hazards regression was conducted to evaluate multivariable-adjusted hazard ratios (HRs) and 95% confidence intervals (CIs) of mortality according to trajectories of quintile of CHEI. Models were first adjusted for age and sex; then additionally adjusted for residence, initial household income changes in household income, changes in marital status, change in PA, change in smoking status, sleep duration, initial BMI, weight change, history of hypertension, and history of diabetes. The changes referred to the ten-year period (1997–2006). In the additional analysis, the Cox proportional hazards regression was used to investigate the shorter-term (taking the last visit of 1997–2006 waves as baseline) and longer-term (taking the first visit of 1997–2006 waves as baseline) associations between baseline quartiles of CHEI and mortality (till 2015), respectively. The adjusted covariates were corresponding baseline values. Mplus (sixth edition; Muthén and Muthén, Los Angeles, CA, USA) was used for LCGA. All other analyses were performed using SAS (version 9.4; SAS Institute Inc., Cary, NC). Statistical significance was defined as *P* < 0.05 (two-sided).

## Results

### Trajectories of CHEI

**Fig. 1:**
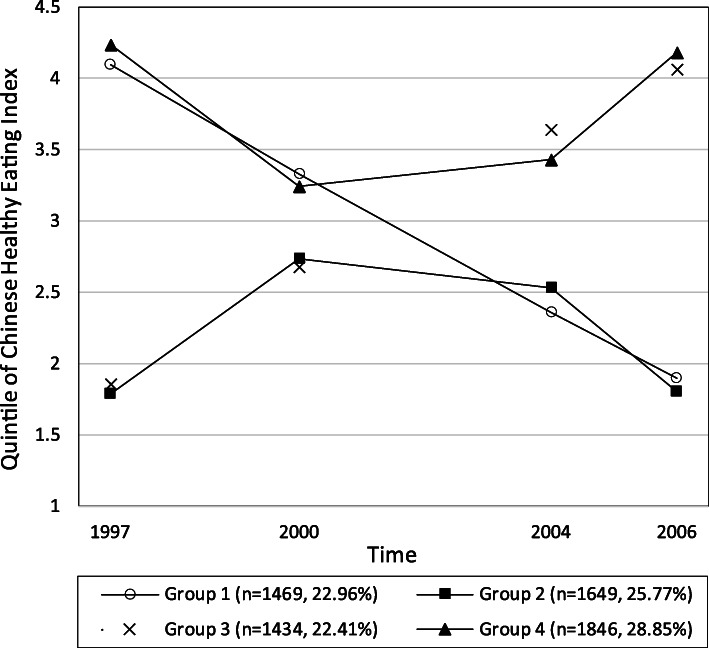
Median of Chinses Healthy Eating Index over waves by identified trajectory groups

After comprehensive evaluation, a four-class solution with quadratic growth parameters specified for the trajectories was identified as the optimal model during the 10-year period. Supplemental Table 2 (Additional file 2) displays the model fit indices for the 2- to 6- class CHEI trajectory models. Figure 1 shows the longitudinal trajectories of median quintiles of CHEI. Group 1 (worsening, *n* = 1469, 22.96%) was characterized by a constant decrease in diet quality over time (highest quintile in 1997 to lowest quintile in 2006). Group 2 (low-moderate-low; *n* = 1649, 25.77%) was characterized by low to moderate diet quality scores over time with the lowest quintiles in 1997 and 2003, Group 3 (improving, *n* = 1434, 22.41%) began with low diet quality in 1997 but diet quality constantly increased over time and scores were in the highest quintile by 2003. Group 4 (high-moderate-high, *n* = 1846, 28.85%) was characterized by moderate to high diet quality with highest scores observed in 1997 and 2003. The plot of absolute value of CHEI showed similar tendency among groups, however, the mean CHEI for all groups across each wave were below 60 (Supplemental Fig. 1 in Additional file 3).

Supplemental Fig. 2 (Additional file 4) shows the proportion of participants meeting the dietary recommendations in DGC-2016 for 17 food components of CHEI and by four trajectories groups, respectively. Food compositions that synchronously changed with the trajectory of CHEI were whole grains and mixed beans, tuber, fruits, dairy, soybeans, fish and seafood, poultry, and seeds and nuts

### Participants’ characteristics by trajectories groups

Participant characteristics and changes in these characteristics of 10 years, according to the trajectory groups are shown in Table 2. Participants in group 2 (low-moderate-low) were older and had a higher proportion of older adults than the other groups. Group 4 (high-moderate-high) were more likely to live in an urban residence and had the highest household income. Participants in group 1 (worsening) and group 2 were less likely to be in a stable marital relationship, that is more people got divorced, widowed, separated, or remain unmarried over time, than the other two groups. Group 1 and group 2 had a higher proportion of participants whose physical activity level remained insufficient throughout the 10 years. Participants in group 2 were less likely to have adequate sleep duration, normal BMI, and incident hypertension. There were no significant differences with regard to sex, smoke status, weight change and history of diabetes among groups.

**Table 2:** Characteristics of participants by trajectories of quintile of Chinese Healthy Eating Index over 10 years (1997–2006, *n* = 6398)

Variable^a^	Trajectories of quintile of Chinese Healthy Eating Index				*P*^b^
	Group 1 (Worsening, *n* = 1469)	Group 2 (Low-moderate-low, *n* = 1649)	Group 3 (Improving, *n* = 1434)	Group 4 (High-moderate-high, *n* = 1846)	
Initial age (year)	43.2 ± 12.7	44.4 ± 14.5	42.1 ± 12.8	42.5 ± 12.1	< 0.001
Old adults, ≥65 years (%)	5.7	10.9	5.2	4.6	< 0.001
Male (%)	48.5	49.4	48.0	49.5	0.81
Urban residence (%)	27.7	28.1	26.6	32.1	0.008
Household income (1 k yuan/year)					
Baseline	27.2 ± 37.7	27.2 ± 58.2	29.8 ± 29.6	31.4 ± 29.3	0.001
Change	8.3 ± 39.7	10.9 ± 58.6	11.2 ± 29.7	11.4 ± 29.3	0.08
Marital status (%)					
Change from married to unmarried	5.4	5.9	4.4	4.3	< 0.001
Remained unmarried	7.4	9.4	7.2	4.9	
Change from unmarried to married or remained married	87.3	84.7	88.4	90.8	
Physical activity (%)^c^					
Remained not enough	45.5	46.6	44.9	40.3	0.008
Change from enough to not	35.6	35.9	36.5	40.0	
Change from not to enough or remained enough	18.9	17.5	18.6	19.7	
Smoke status (%)					
Remained current smoker	30.0	29.8	29.6	28.8	0.43
Change from current smoker to former smoker	10.3	9.3	8.2	8.6	
Remained not current smoker	59.8	60.9	62.3	62.6	
Proper sleep duration (%)^d^	75.6	65.5	71.8	76.6	< 0.001
Initial normal body mass index (%)	21.8	19.6	25.5	27.6	< 0.001
Weight change (kg)	0.8 ± 3.6	0.7 ± 2.1	0.6 ± 2.1	0.7 ± 2.1	0.34
History of hypertension (%)					
Never	66.3	69.5	67.6	65.4	0.04
Incident during the 10-year period	14.5	14.8	16.1	15.6	
Diagnosed from the beginning	19.2	15.7	16.3	19.1	
History of diabetes (%)					
Never	95.5	95.6	95.1	95.8	0.95
Incident during the 10-year period	2.5	2.5	2.4	2.3	
Diagnosed from the beginning	2.0	1.9	2.4	1.9	

^a^Values are mean ± SD for continuous variables and % for categorical variables

^b^Chi-square test for categorized variables and one-way ANOVA test for continuous variables

^c^Enough physical activity was defined as meeting the WHO’s recommendation

^d^Proper sleep duration was defined as meeting the US National Sleep Foundation’s recommendation

### Trajectories of CHEI and mortality

During 29,836.4 person-years of follow-up, a total of 482 or 7.5% deaths were documented. The multivariate cox proportional hazards models showed that the diet quality trajectories of participants in group 3 (improving) and group 4 (high-moderate-high) were associated with lower total mortality when compare with group 2 (low-moderate-low) (Table 3). The HRs and 95% CI were 0.73 (0.55, 0.97) for group 3 and 0.76 (0.59, 0.98) for group 4, respectively. No significant associations with mortality were found for group 1 (worsening), when compared to group 2. When we stratified analyses by participant characteristics, the association between mortality and the trajectories of diet quality differed among sub-population. Generally speaking, the inverse association with trajectories of CHEI in group 3 and group 4 compared with group 2 remained significant only among those who were young adults (20 to < 65 years), males, and urban residents. The magnitude of the inverse association between trajectory of CHEI and mortality was stronger for group 3 than group 4.

**Table 3:** Associations of trajectories of quintile of Chinese Healthy Eating Index during the 10-year period (1997–2006) with the subsequent risk of death in all respondents and subgroups (*n* = 6398)

	Trajectories of quintile of Chinese Healthy Eating Index			
	Group 1 (Worsening)	Group 2 (Low-moderate-low)	Group 3 (Improving)	Group 4 (High-moderate-high)
All respondents				
*N*, death/total	110/1469	178/1649	80/1434	114/1846
Basic model, HR (95% CI)^a^	0.99 (0.78, 1.26)	Ref.	0.80 (0.61, 1.04)	0.79 (0.62, 1.002)
Multivariable model, HR (95% CI)^b^	0.91 (0.71, 1.18)	Ref.	0.73 (0.55, 0.97)^*^	0.76 (0.59, 0.98)^*^
Baseline age				
20 to < 65 years				
*N*, death/total	75/1387	109/1474	54/1361	88/1762
Multivariable model, HR (95% CI)^2^	0.80 (0.59, 1.09)	Ref.	0.62 (0.44, 0.87)^**^	0.74 (0.55, 0.99)^*^
≥ 65 years				
*N*, death/total	35/82	69/175	26/73	26/84
Multivariable model, HR (95% CI)^b^	1.14 (0.70, 1.84)	Ref.	1.02 (0.61, 1.69)	0.85 (0.49, 1.48)
Sex				
Male				
*N*, death/total	60/713	104/815	44/688	73/913
Multivariable model, HR (95% CI)^b^	0.75 (0.53, 1.04)	Ref.	0.58 (0.40, 0.85)^**^	0.61 (0.44, 0.85)^**^
Female				
*N*, death/total	50/756	74/834	36/746	41/933
Multivariable model, HR (95% CI)^b^	1.21 (0.80, 1.81)	Ref.	1.00 (0.64, 1.56)	1.07 (0.70, 1.64)
Baseline residence				
Rural				
*N*, death/total	21/389	50/465	18/398	25/615
Multivariable model, HR (95% CI)^b^	1.25 (0.70, 2.23)	Ref.	0.95 (0.50, 1.80)	0.64 (0.36, 1.13)
Urban				
*N*, death/total	89/1080	128/1184	62/1036	89/1231
Multivariable model, HR (95% CI)^b^	0.87 (0.65, 1.15)	Ref.	0.71 (0.51, 0.98)^*^	0.80 (0.60, 1.08)

^a^ Basic model adjusted for age and sex

^b^ Multivariable model adjusted for age, sex, residence (rural or urban), initial household income and changes in household income (all in quantile, inflated to 2015), changes in marital status, changes in physical activity, changes in smoke status, sleep duration (short, meet the US National Sleep Foundation’s recommendation, long), initial BMI (< 18.5, 18.5–23.9, 24–27.9, or ≥ 28), weight change (in quintiles), history of hypertension, and history of diabetes. The changes referred to the ten-year period (1997–2006)

^*^P < 0.05, ^**^P < 0.01, ^***^P < 0.001

Supplemental Table 3 (Additional file 5) presents the additional analysis of baseline CHEI and mortality. In the shorter-term analysis with 30,672.8 person-years of follow-up, after adjusting for covariates, the HR (95% CI) for mortality was 0.74 (0.56, 0.99), when comparing the highest with the lowest baseline quartiles of CHEI. In the long-term analysis with 83,992.9 person-years of follow-up, the associations were insignificant across quartiles of CHEI

## Discussion

In this large cohort of Chinese adults, we identified four distinct trajectories of quintiles of CHEI over 10 years, from 1997 to 2006. Groups characterized by higher or improving diet quality over time were prospectively associated with lower mortality risk, when compared to those with lower diet quality. Favorable associations were more evident in young adults, males, and urban residents. Differences in diet quality over time between the groups were mostly explained by differences in consumption of whole grains and mixed beans, tuber, fruits, dairy, soybeans, fish and seafood, poultry, and seeds and nuts.

From 1997 to 2006, the Chinese per-capita GDP rose from 781.7 US dollars to 2099.2 US dollars, which covers the inflection point of the growth curve [28]. Following the rapid economic and social change, China has witnessed a significant nutrition transition in the past several decades [13–15]. Our results support other evidence on the transition suggesting that despite some modest improvements, diet quality was still overall suboptimal [15]. The traditional Chinese dietary pattern has been rapidly altered. Specifically, the food consumption has been characterized by declines in intake of cereal, whole grains, and an increase in intake of red meat and processed meat. Although there are several improvements in diet quality, including moderate increased intake of fruits, dairy, fish and seafood, and seeds and nuts, the majority of the population still did not meet the respective dietary recommendations (Supplemental Fig. 3 in Additional file 6). Similar features of the Chinese nutrition transition were also reported in studies with a longer time scale [14, 15].

The disparity of the four identified CHEI trajectory groups may partly be explained by changes in household income. Socio-economic disparities in diet quality has been well studied [29, 30]. On one side, the socio-economic differences in nutrient intake can be substantially explained by the monetary cost of the diet [29]. On the other side, the rapid income growth in China was shown to have adverse effect on diet quality, especially for the poor [30]. With these theories, the curves of median household income over waves should partly explain disparities in the CHEI trajectories (Supplemental Fig. 4 in Additional file 7), where adults with lower income over time experienced worsening diet quality, when compared to adults whose income increased or stayed high.

Few studies have investigated the associations between changes in diet quality and the risk of death, worldwide and in China [31, 32]. Sotos-Prieto et al. studied the changes in diet quality over 12 years (1986–1998) and the risk of death in the subsequent 12 years among US health professionals [31]. Those who had the greatest improvement (13 to 33%) in diet quality had a lower total mortality, as compared with those who had a relatively stable diet quality (0 to 3% improvement): the pooled HRs and 95% CIs were 0.91 (0.85, 0.97) for the AHEI score, 0.84 (0.78, 0.91) for the Alternate Mediterranean Diet (aMED) score, and 0.89 (0.84, 0.95) for the DASH diet score [31]. In a cohort study in Singapore Chinese adults, a 12–20% lower risk of all-cause mortality was observed for highest versus lowest quintiles of baseline (1993–1998) AHEI, aMED, DASH, and Healthy Diet Indicator scores [33]. Gao et al. explored the trajectories of Mediterranean Diet Adherence (MDA) (1997–2011) and the risk of hypertension among Chinese adults [32]. Similar to our findings, people’s diet quality was categorized into groups of initially high, increasing higher, initially low and decreasing lower.

Those scores, however, are based on dietary guidelines designed for western countries and they differed on recommendations for food components like dairy products, meats, and sodium [34]. The DGC was proclaimed by the Chinese Nutrition Society and Ministry of Health and was specifically designed for Chinese people to foster optimal nutrition in the context of nutrition transition [7]. Thus, it is more appropriate to use DGC to examine diet quality in the Chinese population. Yu et al. found that higher baseline adherence to the DGC-2007 was associated with a lower risk of total mortality among Chinese adults in urban Shanghai [34]. The magnitude of association was stronger among males with a mean follow-up of 6.5 years than females with a mean follow-up of 12.0 years [31]. Results of the current study showed that higher baseline diet quality was associated with lower mortality in the shorter-term, but not in the longer-term. It suggests that baseline diet quality may not be a good predictor of long-term mortality, possibly because of changes in diet quality. Another cross-sectional study calculated the PAFs to estimate the proportion of cardiometabolic death attributable to suboptimal dietary intake among Chinese adults in 4 years [15]. The PAFs of a combination of 12 dietary factors based on DGC were 62·2% in 1982, 57.9% in 1992, 56.2% in 2002, and 51.0% in 2010–12 with the absolute number of cardiometabolic deaths substantially increased [15]. Complementarily, results from the current study contribute to the evidence of association between longitudinal change of overall diet quality and mortality using an index based on the DGC.

The relationship between changes in diet quality and mortality may, to a large extent, be driven by chronic diseases like cardiovascular disease, diabetes, and cancer. The research on trajectories of MDA found that Chinese adults with initially high, and increasingly higher, MDA had lower BP and lower risk of developing hypertension [32]. Another longitudinal study found that older adults who were in the highest quintile for traditional Chinese diet had a − 0.23 (95% CI: − 0.44, − 0.02) decrease for BMI, a − 0.90 (95% CI: − 1.42, − 0.37) decrease for weight, and a − 1.57 (95% CI: − 2.32, − 0.83) decrease for waist circumference [35]. In a third cohort study of Chinese adults, people with a healthier initial dietary pattern had lower -lower HbA1c (β = − 1.64, 95% CI: − 3.17, − 0.11) [36]. Moreover, previous studies on changes in diet quality have consistently reported stronger associations with cardiovascular-cause mortality than total mortality [31, 33, 34]. Nevertheless, significant associations with the longitudinal trajectories of diet quality in the current study are independent of the history of hypertension and diabetes and suggests that long-term diet quality is an important predictor of mortality risk.

Nevertheless, the association between diet quality trajectories of participants in the worsening group and mortality was not significant as compared to the low-moderate-low group. It could be that the differences in diet quality may not have been large enough, or the length of follow-up not long enough, to see a significant association. Long-term prospective studies are needed to distinguish the effects of changing diet quality on mortality.

The present study found clear differences in certain sociodemographic characteristics across diet quality trajectory groups. For example, participants in group 1 (worsening) and group 2 (low-moderate-low) were less likely to be in a stable marital relationship, and had worse physical activity level. Participants in group 2 were older and less likely to have adequate sleep duration, normal BMI, and incident hypertension. The present study also found sociodemographic differences for the association between diet quality trajectories and total mortality. A similar sex difference was also reported in a previous study in China, where higher baseline diet quality was associated with a 33% (95% CI: 25, 40%) decrease of mortality in men, whereas a 23% (5, 20%) decease of mortality in women [34]. The wider 95% CI of the HR estimates for women compared to men suggests greater population variability and other explanatory factors that were not accounted for in the present study. The specific cause of death and medication history may result in the sex difference in the association between diet quality trajectories and total mortality. First, there are known sex differences in the components of mortality burden [37]. Globally, men tend to have more hypertension while women tend to have more dyslipidemia and inflammatory-related diseases [37]. Second, research in China showed that women have poorer compliance with mediations and treatment to control CVD risk factors (irrespective of education and area of residence) [38]. The present study lacked information on specific cause of death and medication history; this information should be collected in future research to better understand the sex-specific associations between diet quality and mortality in Chinese adults.

## Strengths and limitations

The strengths of our study include the: prospective study design, use of a diet quality index that reflects adherence to the Chinese Dietary Guidelines, and repeated measurement of diet and important covariates. However, our study also has several limitations. Firstly, dietary intake was based on three consecutive 24 h recalls which are subject to measurement error (e.g., recall bias or systematic misreporting) and may not reflect the usual pattern of dietary intake, particularly for infrequently consumed foods. However, repeat 24-h recalls were still recommended as the most appropriate method for a UK national study of diet in low-income households [39]. Secondly, added sugar intake in the current study might be underestimated due to the lack of data in the Chinese Food Composition, and this issue was acknowledged in the development and validation of CHEI [11, 12]. Despite the potential underestimation of added sugar, the diet quality score, as a comprehensive assessment of 17 components, was well validated [12]. Thirdly, although multiple covariates were adjusted for in the analyses, residual confounding arising from unmeasured/unknown confounders or imprecisely measured confounders cannot be ruled out. Furthermore, the CHNS lacked data for cause-specific mortality which may provide more information for targeted health promotion efforts, and thus merits further research. Also, the inclusion rate in the entire sample was relatively low due to a large proportion of dropouts and the strict inclusion criteria for the study design. This limited the representativeness of the analytical sample and generalizability of the study findings. Finally, the CHNS is a stratified survey of nine provinces (i.e. not a national survey) and participants resided in mainland China, which may limit the generalizability of the findings.

## Conclusions

Using the CHEI to assess dietary in the Chinese population from 1997 to 2006 demonstrated four trajectories of diet quality. Participants in trajectories of initially high or increasing higher CHEI had lower risk of death in the subsequent 9 years. The DGC-2016 should be promoted by policymakers and physicians to foster population health, meanwhile demographic differences should be taken into consideration including age, sex, and residence.

## Supplementary Information

**Additional file 1: Supplemental Table 1**. Comparison of characteristics between included and excluded participants, China Health and Nutrition Survey 1997-2006

**Additional file 2: Supplemental Table 2** Model fit of latent class growth analysis – add quadratic term with correlated errors

**Additional file 3: Supplemental Fig. 1**. Means of Chinses Healthy Eating Index over waves by identified trajectory groups

**Additional file 4: Supplemental Fig. 2**. Proportion of Chinese adults meeting the dietary recommendations for food components by identified trajectory groups

**Additional file 5: Supplemental Table 3**. HR (95% CI) for total mortality by baseline quartiles of Chinese Healthy Eating Index in the shorter- and longer-term (*n* = 6398)

**Additional file 6: Supplemental Fig. 3**. Proportion of Chinese adults meeting the dietary recommendations for 17 food components

**Additional file 7: Supplemental Fig. 4**. Median of household income over waves by identified diet quality trajectory groups

## Data Availability

The datasets generated and/or analyzed during the current study are available in the CHNS website. https://www.cpc.unc.edu/projects/china

## Abbreviations

CHEI: Chinese Healthy Eating Index
HEI: Healthy Eating Index
AHEI: Alternative Healthy Eating Index
DASH: Dietary Approaches to Stop Hypertension
GBD: Global Burden of Disease
PAF: Population Attributable Fraction
CHNS: China Health and Nutrition Survey
SP: Standardized Portion
HR: Hazard Ratios
CI: Confidence Interval
MDA: Mediterranean Diet Adherence
