# Correction to: Longitudinal trajectories of diet quality and subsequent mortality among Chinese adults: results from the China health and nutrition survey 1997–2015

**DOI:** 10.1186/s12966-021-01127-6

**Published:** 2021-04-29

**Authors:** Ming-wei Liu, Sarah A. McNaughton, Qi-qiang He, Rebecca Leech

**Affiliations:** 1 Julius Global Health, The Julius Center for Health Sciences and Primary Care, University Medical Center Utrecht, Utrecht, the Netherlands; 2 School of Health Sciences, Wuhan University, Wuhan, 430071 China; 3 Institute for Physical Activity and Nutrition (IPAN), School of Exercise and Nutrition Sciences, Deakin University, 221 Burwood Highway, Burwood, Victoria 3125 Australia; 4 Hubei Biomass-Resource Chemistry and Environmental Biotechnology Key Laboratory, Wuhan University, Wuhan, 430071 China

**Correction to: Int J Behav Nutr Phys Act 18, 51 (2021)**

**https://doi.org/10.1186/s12966-021-01118-7**

Following publication of the original article [1], the authors identified an error in Fig. 1. The correct figure is given below.

**Fig. 1:**
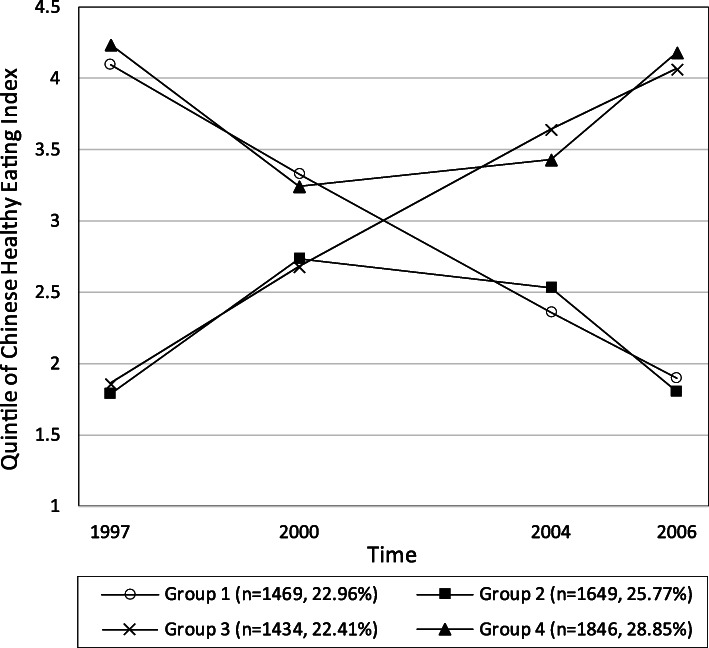
Median of Chinses Healthy Eating Index over waves by identified trajectory groups

The author group has been updated above and the original article [1] has been corrected.
