# Supplementary material for: Longitudinal trajectories of diet quality and subsequent mortality among Chinese adults: results from the China health and nutrition survey 1997–2015
Source: Int J Behav Nutr Phys Act. 2021 Apr 7;18:51. doi: 10.1186/s12966-021-01118-7 (PMC8028751; doi:10.1186/s12966-021-01118-7)
Supplement: Supplementary file 1 — Additional file 1: Supplemental Table 1. Comparison of characteristics between included and excluded participants, China Health and Nutrition Survey 1997-2006 [file 12966_2021_1118_MOESM1_ESM.docx]

**Supplemental Table 1.** Comparison of characteristics between included and excluded participants, China Health and Nutrition Survey 1997-2006

| Baseline characteristics | Included (n=6398, 39.7%) | Excluded (n=9717, 60.3%) | *P*^3^ |
| --- | --- | --- | --- |
| Age (year), median (Q1, Q3) | 43 (34, 52) | 35 (24, 52) | <0.001 |
| Male (%) | 51.1 | 52.0 | 0.25 |
| Urban residence (%) | 29.2 | 38.8 | <0.001 |
| Household income (1k yuan/year), median (Q1, Q3) | 14.8 (8.4, 23.7) | 17.9 (9.5, 29.6) | <0.001 |
| Marital status (%) |  |  |  |
| Never married | 6.5 | 25.3 | <0.001 |
| Married | 88.4 | 66.5 |  |
| Divorced/Widowed/Separated | 5.1 | 8.1 |  |
| Physical activity (%)^1^ |  |  |  |
| No | 29.8 | 42.8 | <0.001 |
| Some but not enough | 23.4 | 18.0 |  |
| Enough | 46.8 | 39.2 |  |
| Smoke status (%) |  |  |  |
| Never | 66.2 | 71.2 | <0.001 |
| Ever smoked | 33.9 | 28.8 |  |
| Proper sleep duration (%)^2^ | 72.1 | 71.1 | 0.21 |
| Body mass index, mean±SD | 22.6±3.1 | 22.6±3.3 | 0.77 |
| History of hypertension (%) | 17.6 | 18.7 | 0.08 |
| History of diabetes (%) | 1.4 | 1.5 | 0.52 |

^1^ Enough physical activity was defined as meeting the WHO’s recommendation.

^2^ Proper sleep duration was defined as meeting the US National Sleep Foundation’s recommendation.

^3^ Tests were Wilcoxon rank sum test for age, household income, and physical activity; t-test for body mass index; and Chi-Square test for other variables.
