# Supplementary material for: Longitudinal trajectories of diet quality and subsequent mortality among Chinese adults: results from the China health and nutrition survey 1997–2015
Source: Int J Behav Nutr Phys Act. 2021 Apr 7;18:51. doi: 10.1186/s12966-021-01118-7 (PMC8028751; doi:10.1186/s12966-021-01118-7)
Supplement: Supplementary file 2 — Additional file 2: Supplemental Table 2 Model fit of latent class growth analysis – add quadratic term with correlated errors [file 12966_2021_1118_MOESM2_ESM.docx]

**Supplemental Table 2.** Model fit of latent class growth analysis – add quadratic term with correlated errors.

|  | 2 classes | 3 classes | 4 classes | 5 classes | 6 classes |
| --- | --- | --- | --- | --- | --- |
| Entropy | 0.633 | 0.688 | 0.668 | 0.649 | 0.695 |
| AIC | 82 572.745 | 82 068.630 | 81 778.964 | 81 704.582 | 81 281.843 |
| Adjusted BIC | 82 622.949 | 82 133.178 | 81 857.856 | 81 797.818 | 81 389.423 |
| Class comparison | 1 v 2 | 2 v 3 | 3 v 4 | 4 v 5 | 5 v 6 |
| H0 Loglikelihood value | -41 272.373 | -41 016.315 | -40 867.482 | -40 826.291 | -40 610.921 |
| VLMR LRT P value of difference | <0.0001 | <0.0001 | <0.0001 | 0.0551 | <0.0001 |
