# Supplementary material for: Longitudinal trajectories of diet quality and subsequent mortality among Chinese adults: results from the China health and nutrition survey 1997–2015
Source: Int J Behav Nutr Phys Act. 2021 Apr 7;18:51. doi: 10.1186/s12966-021-01118-7 (PMC8028751; doi:10.1186/s12966-021-01118-7)
Supplement: Supplementary file 4 — Additional file 4: Supplemental Fig. 2. Proportion of Chinese adults meeting the dietary recommendations for food components by identified trajectory groups [file 12966_2021_1118_MOESM4_ESM.docx]

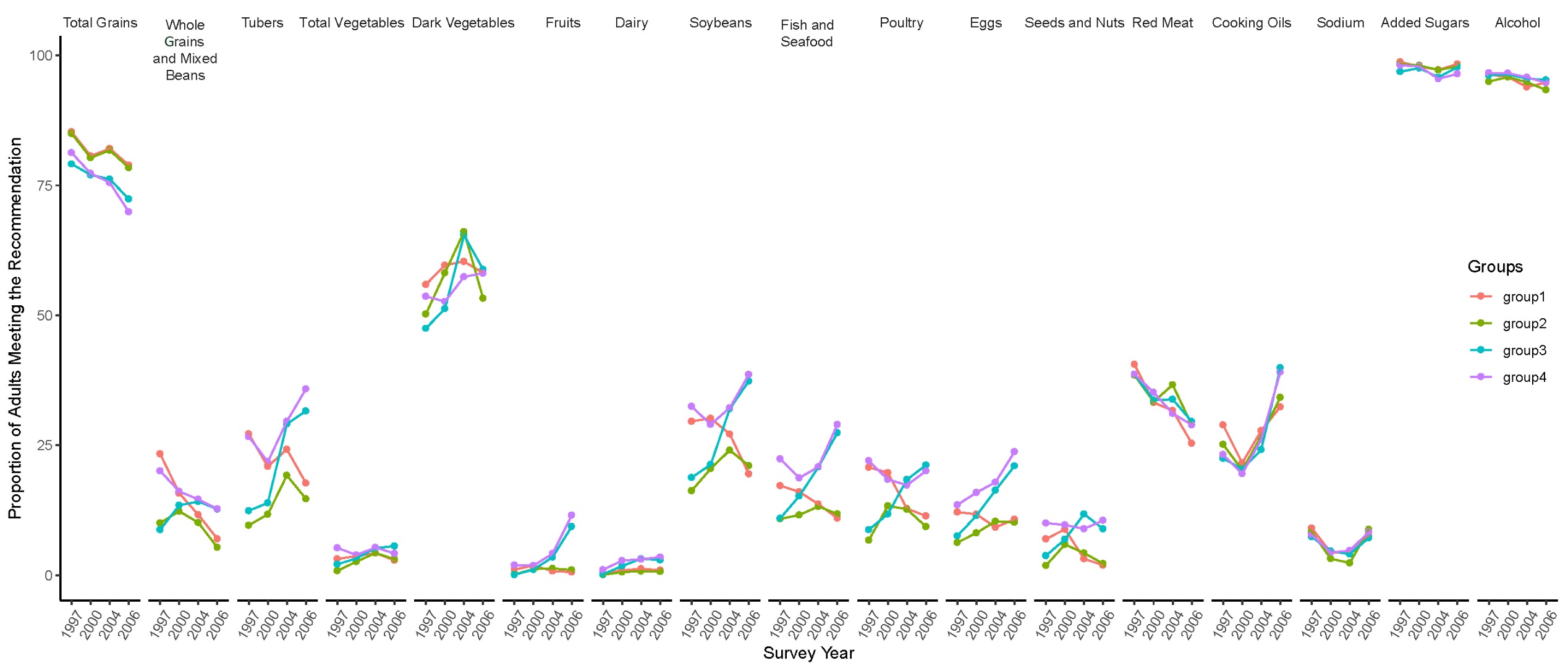


**Supplemental Fig. 2** Proportion of Chinese adults meeting the dietary recommendations for food components by identified trajectory groups
