# Supplementary material for: Longitudinal trajectories of diet quality and subsequent mortality among Chinese adults: results from the China health and nutrition survey 1997–2015
Source: Int J Behav Nutr Phys Act. 2021 Apr 7;18:51. doi: 10.1186/s12966-021-01118-7 (PMC8028751; doi:10.1186/s12966-021-01118-7)
Supplement: Supplementary file 5 — Additional file 5: Supplemental Table 3. HR (95% CI) for total mortality by baseline quartiles of Chinese Healthy Eating Index in the shorter- and longer-term (n = 6398) [file 12966_2021_1118_MOESM5_ESM.docx]

**Supplemental Table 3.** HR (95% CI) for total mortality by baseline quartiles of Chinese Healthy Eating Index in the shorter- and longer-term^1^ (n=6398)

|  | Quartile of Chinese Healthy Eating Index | | | | *P* trend |
| --- | --- | --- | --- | --- | --- |
|  | 1 | 2 | 3 | 4 |  |
| Shorter-term |  |  |  |  |  |
| *N*, death/total | 154/1597 | 133/1606 | 111/1597 | 84/1598 |  |
| Basic model^2^ | Ref. | 0.88 (0.69, 1.11) | 0.88 (0.68, 1.12) | 0.76 (0.58, 0.99)^*^ | 0.05 |
| Multivariate model^3^ | Ref. | 0.81 (0.64, 1.04) | 0.80 (0.62, 1.03) | 0.74 (0.56, 0.99)^*^ | 0.03 |
| Longer-term |  |  |  |  |  |
| *N*, death/total | 123/1570 | 139/1567 | 122/1594 | 98/1667 |  |
| Basic model^2^ | Ref. | 1.14 (0.89, 1.46) | 1.09 (0.85, 1.41) | 0.98 (0.75, 1.28) | 0.87 |
| Multivariate model^3^ | Ref. | 1.18 (0.89, 1.55) | 0.99 (0.74, 1.33) | 1.03 (0.74, 1.44) | 0.80 |

^1^ The shorter-term model took the last visit of the 1997-2006 waves as the baseline and track the mortality status till 2015. The longer-term model took the first visit of the 1997-2006 waves as the baseline and track the mortality status till 2015.

^2^ Basic model adjusted for age and sex.

^3^ Multivariable model adjusted for baseline age, sex, residence (rural or urban), household income (in quantile, inflated to 2015), marital status (Never married, Married, Divorced/Widowed/Separated), physical activity (No, Some but not enough, Meeting the WHO’s recommendation), smoke status, sleep duration (short, meet the US National Sleep Foundation’s recommendation, long), BMI, history of hypertension, and history of diabetes.

^*^P< 0.05, ^**^P<0.01, ^***^P<0.001
